# Supplementary material for: The ER tether VAPA is required for proper cell motility and anchors ER-PM contact sites to focal adhesions
Source: eLife. 2024 Mar 6;13:e85962. doi: 10.7554/eLife.85962 (PMC10917420; doi:10.7554/eLife.85962)

Figure 1_Original blots

Blots presented in Figure 1A

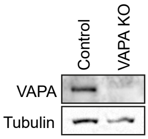

Original blot for VAPA

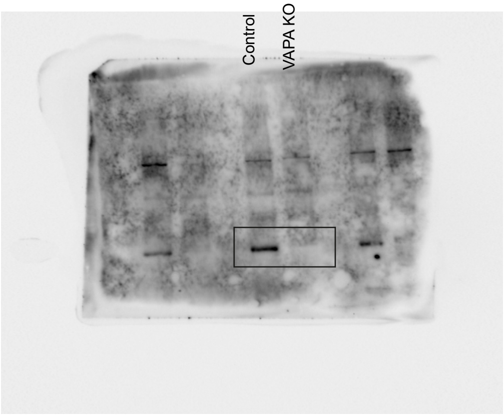

Original blot for Tubulin

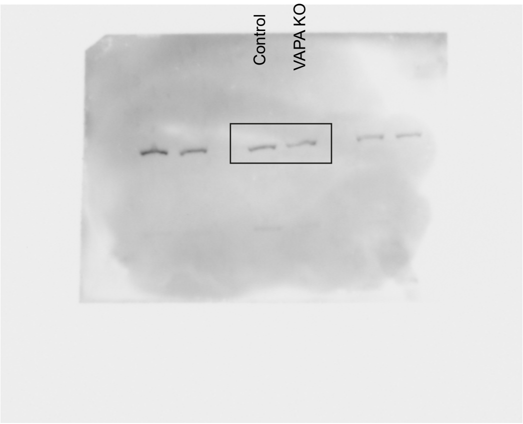

Supplement: Figure 1—source data 2. [file elife-85962-fig1-data2.zip › Figure 1_OriginalBlots.pdf]
