# Supplementary figures and images for: The ER tether VAPA is required for proper cell motility and anchors ER-PM contact sites to focal adhesions
Source: eLife. 2024 Mar 6;13:e85962. doi: 10.7554/eLife.85962 (PMC10917420; doi:10.7554/eLife.85962)

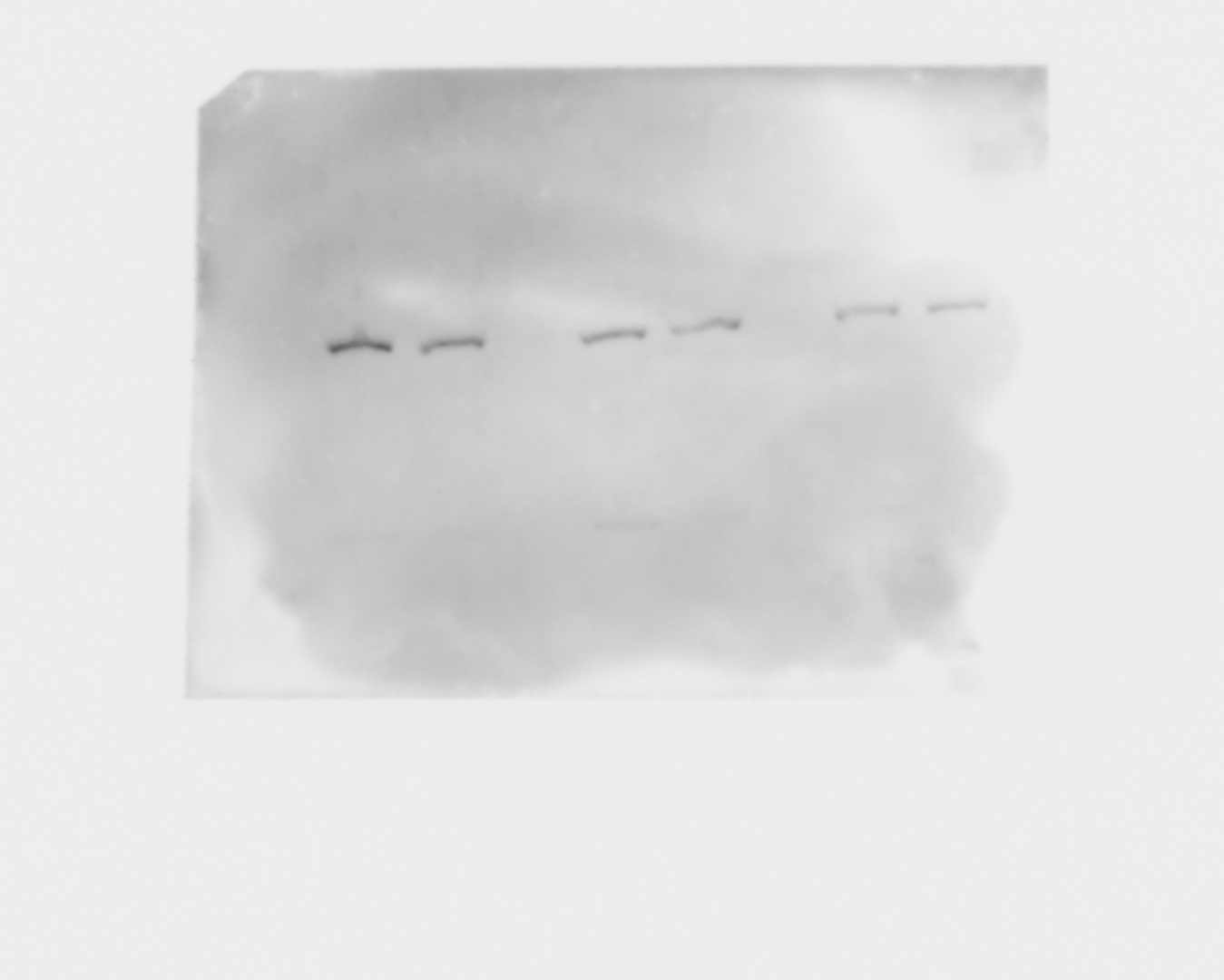

Supplement: Figure 1—source data 2. [file elife-85962-fig1-data2.zip › Fig1_OriginalBlot_Tubulin.tif]

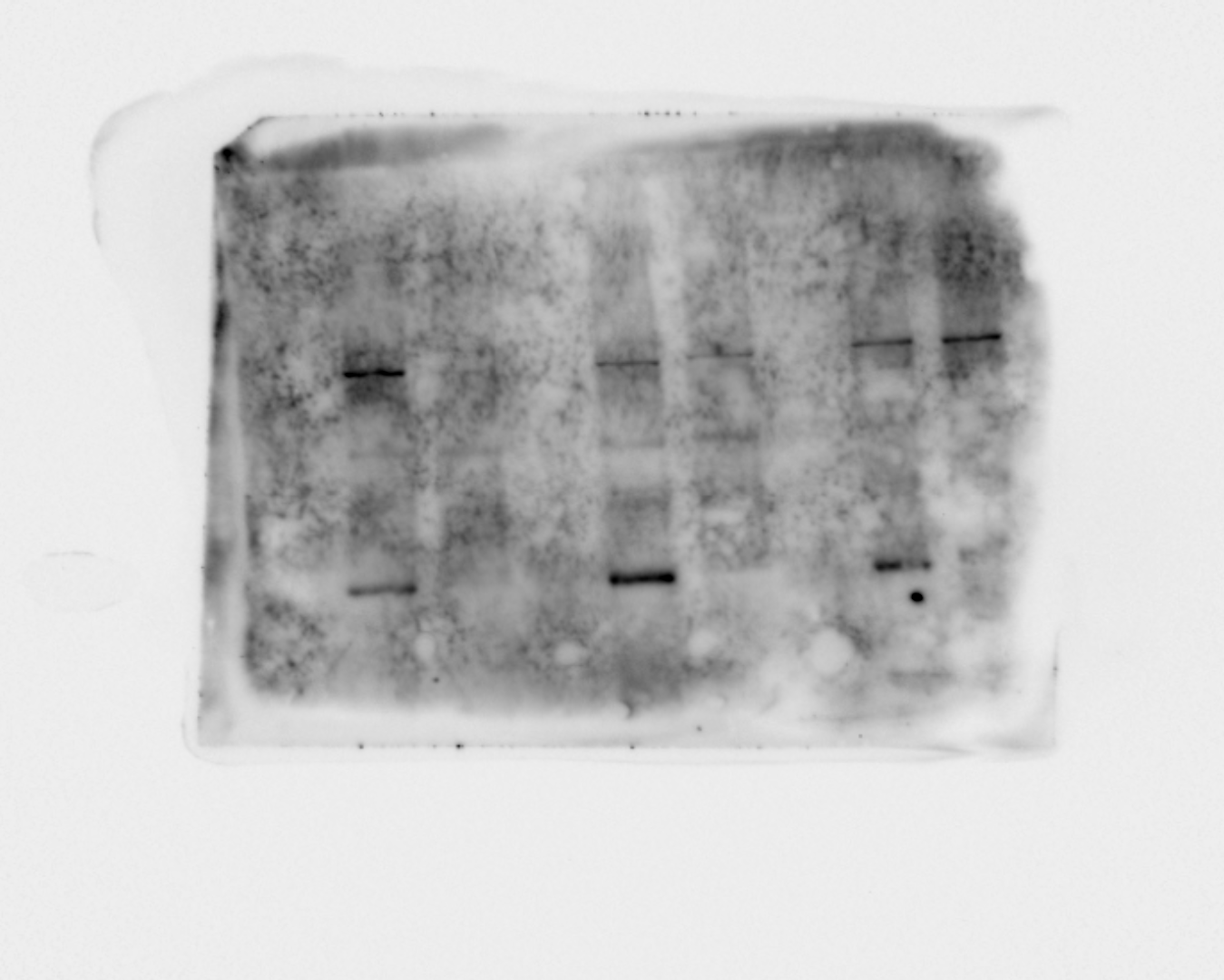

Supplement: Figure 1—source data 2. [file elife-85962-fig1-data2.zip › Fig1_OriginalBlot_VAPA.tif]

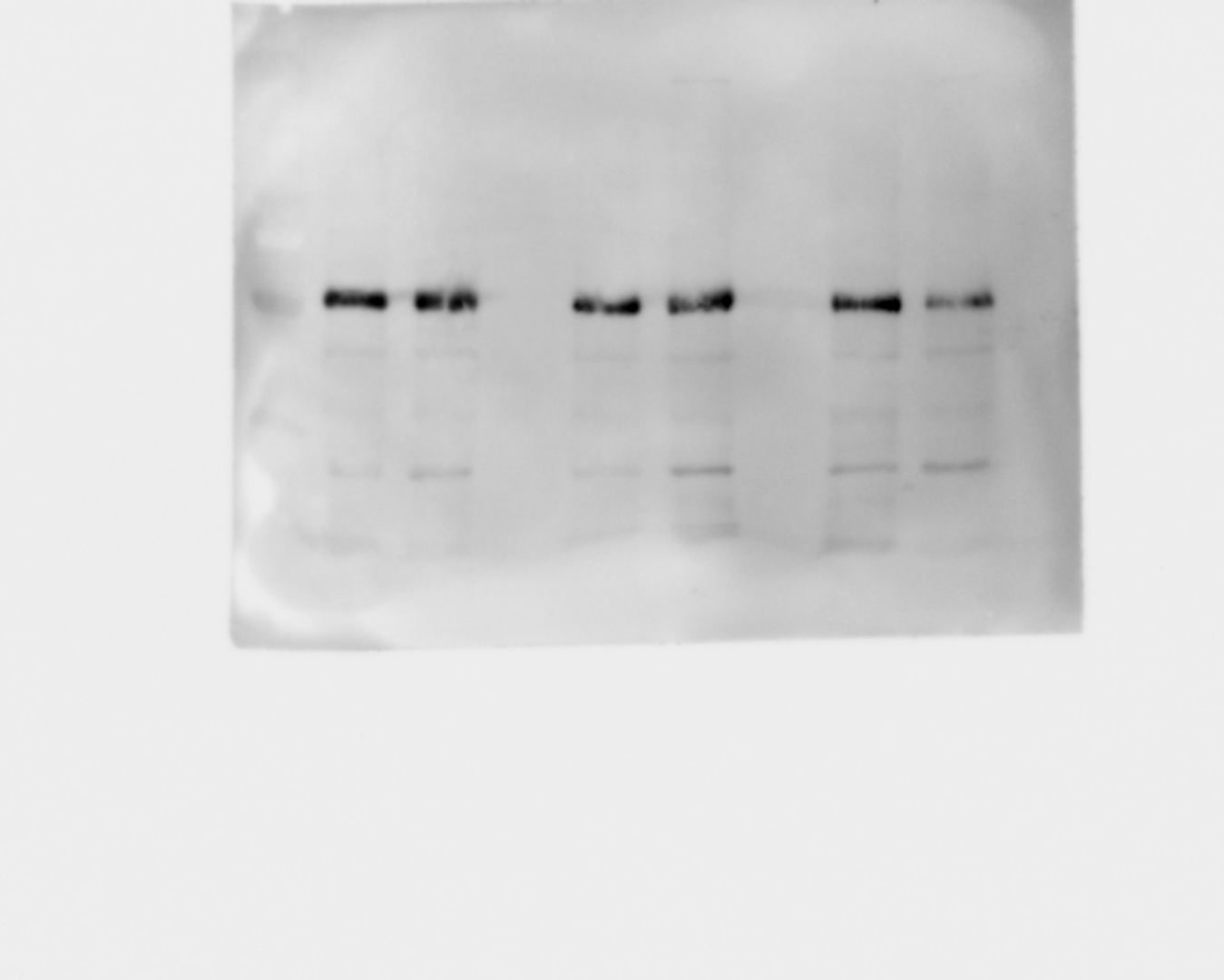

Supplement: Figure 1—figure supplement 1—source data 2. [file elife-85962-fig1-figsupp1-data2.zip › Fig1-figSup1_OriginalBlot_Tubulin.tif]

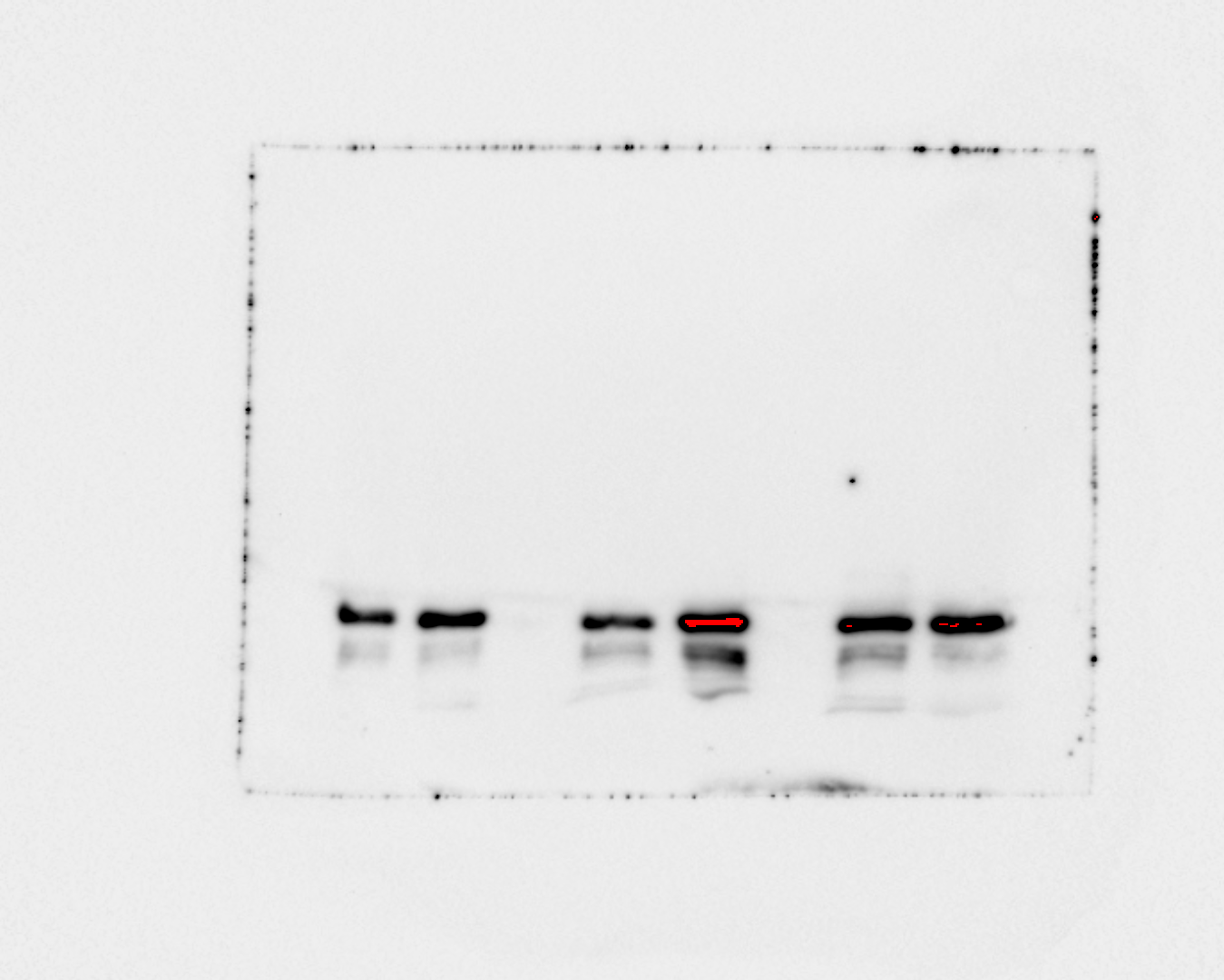

Supplement: Figure 1—figure supplement 1—source data 2. [file elife-85962-fig1-figsupp1-data2.zip › Fig1-figSup1_OriginalBlot_VAPB.tif]

Blots presented in Figure 1A

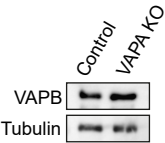

Original blot for VAPB

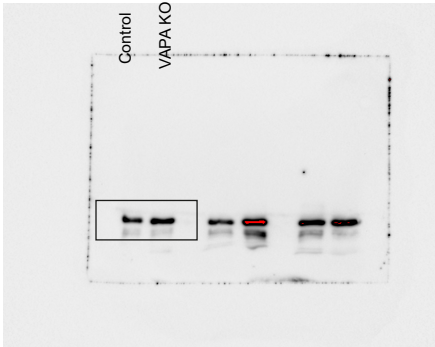

Original blot for Tubulin

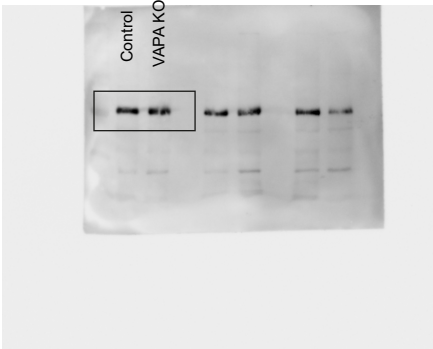

Supplement: Figure 1—figure supplement 1—source data 2. [file elife-85962-fig1-figsupp1-data2.zip › Figure 1-figSup1_OriginalBlots.pdf]
